# Optimizing congenital cytomegalovirus detection by pool testing in saliva by a rapid molecular test

**DOI:** 10.1007/s00431-023-05183-x

**Published:** 2023-09-09

**Authors:** Giannina Izquierdo, Mauricio J Farfan, Leonel Villavicencio, Luisa Montecinos, Felipe Tarque, William Acevedo, Roberto Reyes, Carolina Guerra, Leslie Araya, Belén Sepúlveda, Camila Cabrera, Pamela Medina, Jocelyn Mendez, Elieder Mardones, Juan P Torres

**Affiliations:** 1 Faculty of Medicine, Universidad de Chile, Department of Pediatrics, Santiago, Chile; 2 Hospital Barros Luco Trudeau, Neonatal Intensive Care Unit, Santiago, Chile; 3 Hospital Lucio Cordova, Molecular Biology Laboratory, Santiago, Chile; 4 Hospital Luis Calvo Mackenna, Molecular Biology Laboratory and Department of Pediatrics, Santiago, Chile; 5 Hospital Exequiel González Cortés, Division of Pediatric Infectious Diseases, Santiago, Chile

**Keywords:** Congenital cytomegalovirus, Screening, Pool-testing, Loop-mediated isothermal amplification (LAMP), Saliva

## Abstract

Universal congenital cytomegalovirus (cCMV) screening in saliva is increasingly recommended. The aim of our study was to correlate the performance of a *point-of-care* rapid molecular test with CMV real time PCR (CMV RT-PCR) detection, using saliva pool-testing in newborns under a universal screening strategy. Saliva swabs were prospectively collected from newborns < 21 days old and tested by Alethia-LAMP-CMV assay in pools of 5 samples. In positive pools, subjects were tested individually and by saliva and urine CMV RT-PCR. A subset of negative pools were studied with both techniques and viral loads in whole blood were determined in positive patients. From 1,642 newborns included in 328 pools, 8 were confirmed by urine CMV RT-PCR, (cCMV prevalence 0,49%). The PPA and NNA of the pooled saliva Alethia-LAMP-CMV testing were 87,5% and 99,8% with a negative and positive predictive value of 99,9% and 77,7%, respectively. Two false positives were detected (0,12%). A subset of 17 negative pools (85 samples), studied by saliva CMV RT-PCR, showed 100% concordance.

*Conclusion*: CMV pool-testing using a rapid molecular test in saliva proved feasible when compared to PCR gold standards. This strategy could improve cost-effectiveness for cCMV universal neonatal screening, based on the low prevalence of the infection and could be a more affordable approach in less developed regions with reduced detection capacity.

**Table:** 

**What is Known:** *• cCMV is the most frequent congenital infection and a leading nongenetic cause of sensorineural hearing loss and brain disease.* * • Universal screening could allow early detection of congenitally infected infants, improving clinical outcome. * * • Saliva PCR is the preferred and non-invasive test for newborn cCMV screening. *	
**What is New:** * • The feasibility of a universal cCMV screening by pool-testing in saliva using a rapid test in pools of 5 samples. * * • PPA and NPA were 87,5 and 99,8% compared to CMV PCR in urine. * * • This strategy could be relevant specially in LMIC where detection capacity is reduced and could improve cost-effectiveness. * * • cCMV prevalence in our center was 0,49%. *

**What is Known:**

*• cCMV is the most frequent congenital infection and a leading nongenetic cause of sensorineural hearing loss and brain disease.*

*
• Universal screening could allow early detection of congenitally infected infants, improving clinical outcome.
*

*
• Saliva PCR is the preferred and non-invasive test for newborn cCMV screening.
*

**What is New:**

*
• The feasibility of a universal cCMV screening by pool-testing in saliva using a rapid test in pools of 5 samples.
*

*
• PPA and NPA were 87,5 and 99,8% compared to CMV PCR in urine.
*

*
• This strategy could be relevant specially in LMIC where detection capacity is reduced and could improve cost-effectiveness.
*

*
• cCMV prevalence in our center was 0,49%.
*

## Introduction

Congenital cytomegalovirus (cCMV) is the most common congenital viral infection with an estimated prevalence of 0.2%–5% worldwide [1–4]. Despite their clinical relevance, cCMV often is not detected because most infected infants are asymptomatic at birth, the unavailability and costs of diagnostic techniques, and/or because screening programs have not been substantially implemented.

Newborn CMV screening allows early detection and eventual treatment to improve clinical outcomes, but the best strategy remains uncertain. However, universal cCMV screening is increasingly recommended [5, 6]. Saliva is an easy-to-obtain sample for CMV testing, as high titers of CMV shed in the saliva of infected newborns have been described [7]. This specimen appears to be an adequate and less invasive sample for newborn screening, however positive saliva results must be confirmed by urine PCR within 3 weeks of age.

Some rapid molecular diagnostic techniques have shown high sensitivity and specificity in newborns for CMV detection. The Alethia-LAMP-CMV^®^ amplification assay is a point of care loop-mediated isothermal amplification (LAMP) technology with the ability to provide results in less than an hour and has reported 100% and 99.8% positive and negative agreement with saliva real-time PCR (CMV RT-PCR), respectively [8]. This technique may be useful for the diagnosis of cCMV in a more rapid manner and with a lower level of infrastructure requirements.

While CMV RT-PCR and LAMP-based assays have become a common diagnostic tool in clinical microbiology, the cost limits their use, especially in clinical scenarios with high volume testing. Pool-testing, which allow screening multiple samples in groups, has the potential to reduce costs while still providing accurate results while awaiting for less expensive methods [9].

The aim of this study was to validate and correlate the performance of a *point-of-care*, rapid molecular CMV test (Alethia-LAMP-CMV^®^ amplification assay) with CMV RT-PCR detection in saliva pools, from newborns under a cCMV universal screening strategy.

## Methods

From September 2022 to May 2023, saliva swabs were prospectively collected with nylon flocked swabs (Copan FLOQSwabs, USA), 1 h after breastfeeding, from preterms and term newborns less than 21 days old born at the Maternity Ward of the Hospital Barros Luco, Santiago, Chile. Children referred from other hospitals after birth were excluded.

**Fig. 1:**
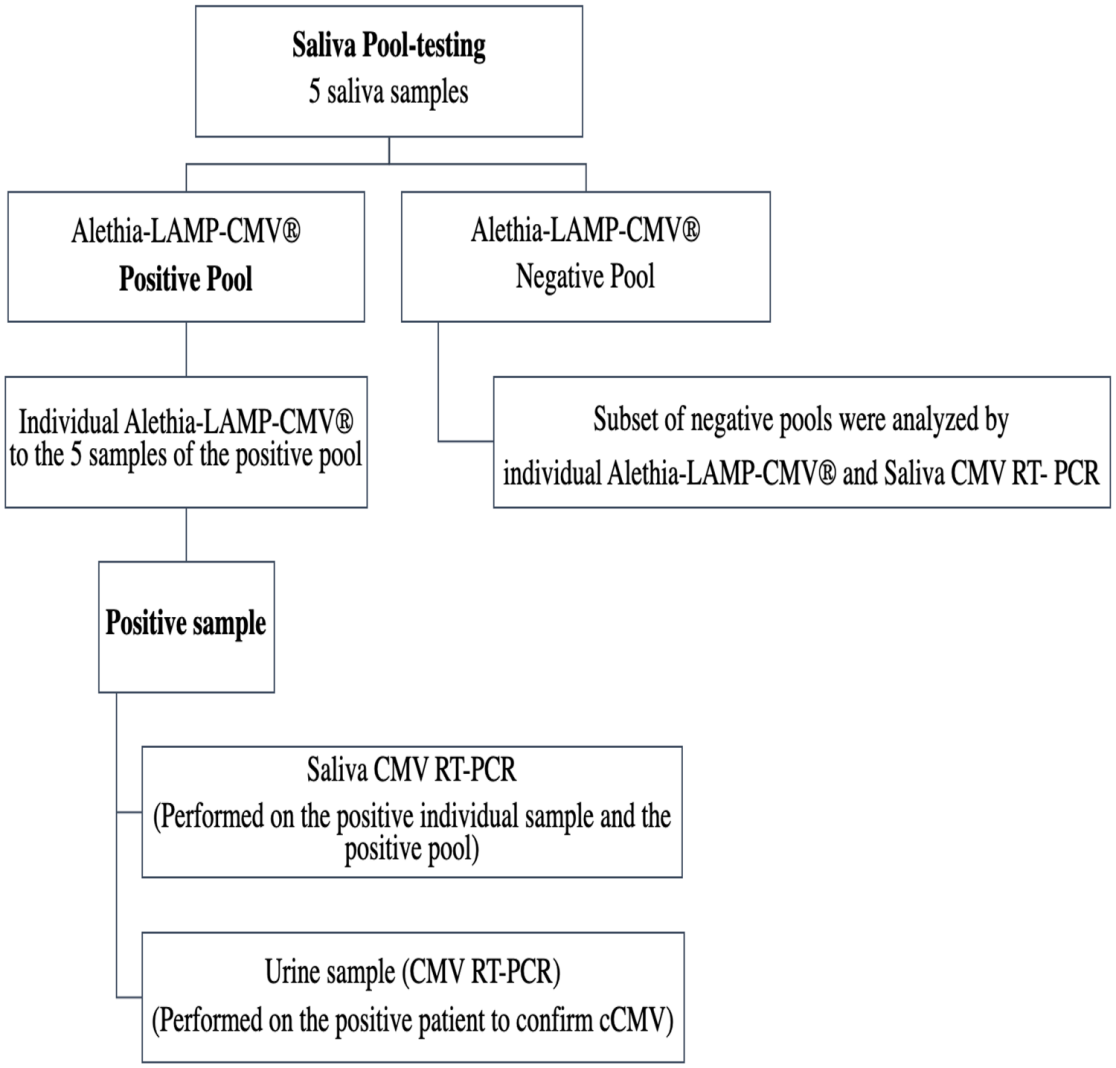
Algorithm used for identification of CMV DNA in pools of 5 saliva samples with a rapid molecular diagnostic test

We performed a rapid molecular test for CMV detection in saliva (Alethia-LAMP-CMV^®^ assay) [10] in pools of 5 samples. In positive pools, samples were tested individually with both, Alethia-LAMP-CMV assay and saliva CMV RT-PCR (TibMolbiol LightMix^®^ Assay kit) [11]. A subset of 17 negative pools and their individual samples were also studied with the rapid molecular test and saliva CMV RT-PCR. All positive cases in saliva were confirmed by CMV RT-PCR in urine samples (Fig. 1) [12, 13].

All newborns with cCMV risk factors (small for gestational age (SGA) [12, 14], infants who failed hearing screening [13, 15], HIV-exposed [14, 16] and < 1.000 gr of birth weight) [13] were evaluated in our center with urine CMV RT-PCR. During the study period, this group was evaluated with both techniques.

### Saliva sample

To collect the specimen, a nylon flocked swab was placed on the inner surface of both infants’ cheeks for 30 s or until the tip appeared saturated. Then, the swab was transferred into a sterile dry tube. Saliva samples were processed at the Molecular Biology laboratory of Hospital Lucio Córdova within the first 24 h after collection if they were stored at room temperature or within 7 days if they were stored at 2 to 8 °C [17].

### Urine samples

The urine sample was collected using pediatric urine collector bags, then placed in 50-mL flasks and stored at 4 °C until processing.

### CMV molecular detection

For detection by the rapid molecular technique in saliva, we use the Alethia-LAMP-CMV^®^ assay, according to the manufacturer's instructions [10]. This technique does not require prior extraction of nucleic acids from the sample. For CMV RT-PCR, nucleic acids extraction of 150 µl of the same pool of samples and the individual samples was performed using MagDEA^®^ Dx SV [18] (automated extraction) on the MagLEAD instrument. The elution volume was set to 50 µl. To detect and quantify viral loads of CMV in saliva and whole-blood, TibMolbiol LightMix^®^ Assay kit [19] and GeneProof Cytomegalovirus^®^ PCR Kit [20], respectively, was performed according to the manufacturer's instructions. CMV RT-PCR on urine samples were qualitatively analyzed by GeneProof Cytomegalovirus^®^ PCR Kit [13, 20].

### Pooling samples

Each dry swab was vortexed for 3–5 s in 500 uL of saline solution. For pooling, 50-µl of 5 saliva samples were used to create 250-µl pools. 100 µl from the pool solution were added to the tube labeled buffer 1, vortexed for 3–5 s, and incubated for 3 min at room temperature. Fifty microliters of buffer 1 was then added to the tube labeled buffer 2 and vortexed for 5 s. Fifty microliters of buffer 2 were transferred to both the test and control chambers of the Alethia test device, which were then placed in the Alethia instrument. Each batch of specimens was run with external controls according to the manufacturer’s instructions [10]. The processing time was 40 min. If the pool was positive, the same procedure was repeated for each individual sample.

### Sample size estimation and statistical analysis

Sample size was determined based on the study by Gantt et al. [8]. A minimum of 1,000 negative prospective CMV samples and a minimum of 5 positive prospectively collected samples were required to be tested to achieve the prespecified Negative Percent Agreement (NPA) of 95%, with a lower 95% confidence interval bound of 85% and to achieve 95% Positive Percent Agreement (PPA), with a lower 95% CI bound of 85%, respectively. CMV prevalence and 95% confidence interval (CI) were calculated for the study population and the high-risk group. Statistical analyses were performed using GraphPad Prism version 6.0 software (La Jolla, CA, USA), considering a p-value of < 0.05 as statistically significant.

## Results

One thousand six hundred forty two out of 2,297 (71,5%) neonates born during the study period were included and screened in 328 saliva pools. Eight newborns were confirmed as true positive cCMV cases by urine CMV RT-PCR (Table 1), of which seven resulted positive by both, pooled and individual saliva Alethia-LAMP-CMV^®^ testing and saliva CMV real-time PCR. Compared to urine CMV RT-PCR, the rapid test showed a Positive Percent Agreement (PPA) of 87,5% (95% CI 47–99%), Negative Percent Agreement (NPA) of 99,8% (95% CI 99,5 -99,9%), and a negative and positive predictive value of 99,9% and 77,7%, respectively. The cCMV prevalence rate of our cohort was 0.49% (95% CI 0,21–0,96%) and the mean age at diagnosis was 2,5 days (SD + 1,4 days). There were two false-positive results reported (0.12%), with positive results in both the Alethia-LAMP-CMV^®^ for the pooled sample and for the individual saliva sample. The CMV-RT PCR tests for the saliva in the pooled sample and individual samples were negative, as was the urine CMV RT-PCR.

**Table 1:** cCMV newborns diagnosed by universal screening with pool-testing in saliva and confirmed by urine CMV RT-PCR

	Alethia-LAMP-CMV pool	Individual Alethia-LAMP-CMV assay	Saliva CMV RT-PCR	Viral Load in saliva (Copies/mL)	Urine CMV RT-PCR*	Viral Load in blood (Copies/mL)	Risk Factor Routine screening
Case 1	Positive	Positive	Positive	19,100 Log 4.28	Positive	7,160 Log 3.85	No
Case 2	Positive	Positive	Positive	5,800 Log 3.76	Positive	66,800 Log 4,82	Yes SGA < p3
Case 3	Positive	Positive	Positive	456,500 Log 5.65	Positive	< 35 Log 1,54	No
Case 4	Positive	Positive	Positive	705,000 Log 5.84	Positive	1,260 Log 3.01	Yes < 1,000 gr
Case 5	Positive	Positive	Positive	3.240.000 Log 6.51	Positive	73 Log 1.86	No
Case 6	Positive	Positive	Positive	71,500,000 Log 7.85	Positive	1,350 Log 3,13	No
Case 7	Positive	Positive	Positive	24,300,000 Log 7.38	Positive	405 Log 2,61	No
Case 8	**Negative**	**Negative**	**Negative**	**Negative**	Positive	80 Log 1,9	Yes SGA < p3 and failure of hearing screening

Risk Factor Routine screening: CMC urine PCR in all small for gestational age (SGA), infants who failed hearing screening, HIV-exposed and < 1.000 gr)/ *Gold standard technique for cCMV diagnosis

In the subset of 17 saliva negative pools (85 individual samples), we observed a 100% of concordance between the rapid test and saliva CMV RT-PCR, in the individual and pool samples.

Out of the total group (n = 1642), 119 (7.2%) corresponded to neonates with cCMV risk factors. In addition to universal screening with the saliva pools, we also screened these newborns with cCMV risk factors with urine CMV RT-PCR. Three subjects in this group were confirmed as true cCMV cases, with positive RT-PCR in urine. The cCMV prevalence in the risk factor group was 2,5% (95% CI 0,52%–7,19%). The pool testing in saliva, individual Alethia-LAMP-CMV^®^ assay, and urine CMV RT-PCR were negative in 116 infants. One SGA newborn, presenting hearing loss screening failure, had a positive urine CMV RT-PCR, but the pool testing and individual Alethia-LAMP-CMV^®^, as well as the saliva RT-PCR were negative (Table 1, true positive case 8). In the cCMV risk factors group, the PPA was 66,6% (IC95 9,43%–99,16%) and the NPA 100% (96,87%–100%) compared with urine CMV RT-PCR with a negative and positive predictive value of 99,15% and 100% respectively.

## Discussion

In the present study we were able to demonstrate that a universal strategy for cCMV using pool testing in saliva samples through a rapid, point-of-care molecular test is feasible. The PPA and NPA of the method were 87,5% and 99,8%, respectively. There were only 2 false positives and only one false negative result, showing a high correlation with the results of CMV RT-PCR in saliva and urine. Interestingly, 5 of 8 of the cCMV cases detected in the study (62.5%) were newborns that would not have been studied for CMV under normal conditions, since they did not correspond to the definition of risk group.

Saliva RT-PCR has proven to have high sensitivity and specificity and is proposed as a potential screening test for CMV infection [21]. On the other hand, pooling saliva samples to study cCMV has shown accuracy in previous studies [9, 22, 23], with the potential to reduce costs and turnaround time. New point-of-care platforms for cCMV diagnosis allow to advance screening programs in centers where molecular biology laboratories are not available, mainly in low-income countries settings [8].

Concerns about false positive results with saliva samples can occur because of virus transmission during breastfeeding. Despite this limitation, the saliva sample is quicker and easier to collect, is less uncomfortable for infants than urine, and thus could be more suitable for a universal screening strategy. Healthcare professionals can be educated to obtain the saliva sample prior to breastfeeding; however, it is not always possible to decrease false positives and parental anxiety.

The two false-positive cases of the rapid test had a negative CMV RT-PCR in saliva, so the viral load could not be measured; while all the true positives cases had high levels of CMV-DNA detected in saliva samples (range Log 3,76–7,85 copies/mL). These findings are in concordance with data reported by Chiereghin et al. concluding that viral load measurement in saliva samples could be useful to discriminate between true-positive and false-positive results. False-positive results were associated with low viral loads (< 2.59 log10 IU/ml), whereas only elevated DNA levels were found in true-positive samples [6]. We propose a pool testing strategy in saliva due to the high titers of CMV shed by infants congenitally infected with the virus.

Seven of the 8 true positive cases in our cohort were detected by positive results in saliva samples, showing saliva viral loads above Log 3,5 copies/mL. Only one subject was a false negative case by the saliva sample, presenting a negative result in the pooled and individual rapid test and in the saliva CMV RT-PCR. This case was detected by a positive urine CMV RT-PCR, performed because he met the CMVc risk group criteria. Interestingly, all confirmed cCMV cases had positive whole-blood viral loads (Table 1), demonstrating a significant robustness of the results.

Our results may have implications for universal cCMV screening strategies. To our knowledge, this is the first report that studies and validates pool testing using a rapid molecular method.

Our study has several limitations. It was conducted in only one hospital center, which despite being one of the largest maternity hospitals in the country (3900 live newborns per year), may not be representative of the entire national population. However, it was performed prospectively and included 71,5% of all newborns in the hospital. We included 5 samples in each pool which seems adequate for a universal screening strategy. This could be a limitation, as the turnaround time also depends on the number of daily child births in a given center. In our context, a pool of 5 saliva samples is optimal. Since cCMV is a low prevalence infection the pool could be further expanded. It is possible that the results could be similar if the pool size is increased to 10 samples, however, further studies would be needed to validate this accordingly. Another limitation may be that the Alethia-LAMP-CMV^®^ assay does not provide quantitative results and is not linked to the Laboratory Information System (LIS). However, the quantitative result is not necessary for the diagnosis of cCMV and the instrument can be connected to the LIS if a special modem is implemented separately. In the present study we report PPA and NPA, rather than sensitivity and specificity of the rapid molecular test. As described by other authors [8, 9], to estimate the PPA and NPA of the rapid test in comparison with urine CMV RT-PCR, we had to assumed a negative result for all urine samples, although we performed both techniques on a subset of 85 samples. Finally, we did not perform an economic or cost-utility evaluation of the pool testing strategy, however, the usefulness of this strategy seems to be better in terms of costs-savings in reagents, execution time and the volume of newborns studied, without an important reduction in sensitivity. Based on our results, we estimate that screening costs can be reduced by four times by pooling five saliva samples, but further studies are necessary to confirm this statement.

In conclusion, CMV pool testing by a rapid molecular test in saliva was feasible to perform in newborns under a universal screening. A high concordance was observed in positives and negatives samples. This strategy could be a new and more cost-effective alternative for cCMV universal neonatal screening due to the low-prevalence nature of this infection and could be a more affordable approach in less developed regions with reduced detection capacity.

## Data Availability

The data from this study can be available upon request to Dr. Giannina Izquierdo (gizquierdo@uchile.cl) or Dr. Juan Pablo Torres (jptorres@uchile.cl).

## References

[1] Manicklal S, Emery VC, Lazzarotto T, Boppana SB, Gupta RK (2013). The "silent" global burden of congenital cytomegalovirus. Clin Microbiol Rev.

[2] Blazquez-Gamero D, Soriano-Ramos M, Vicente M, Pallas-Alonso CR, Perez-Rivilla A, Garcia-Alvarez M (2020). Prevalence and Clinical Manifestations of Congenital Cytomegalovirus Infection in a Screening Program in Madrid (PICCSA Study). Pediatr Infect Dis J.

[3] Rawlinson WD, Boppana SB, Fowler KB, Kimberlin DW, Lazzarotto T, Alain S (2017). Congenital cytomegalovirus infection in pregnancy and the neonate: consensus recommendations for prevention, diagnosis, and therapy. Lancet Infect Dis.

[4] Ssentongo P, Hehnly C, Birungi P, Roach MA, Spady J, Fronterre C et al (2021) Congenital Cytomegalovirus Infection Burden and Epidemiologic Risk Factors in Countries With Universal Screening: A Systematic Review and Meta-analysis. JAMA Netw Open 4(8):e2120736. 10.1001/jamanetworkopen.2021.20736 10.1001/jamanetworkopen.2021.20736 PMC8383138 34424308

[5] Ronchi A, Shimamura M, Malhotra PS, Sanchez PJ (2017). Encouraging postnatal cytomegalovirus (CMV) screening: the time is NOW for universal screening. Expert Rev Anti Infect Ther.

[6] Chiereghin A, Pavia C, Turello G, Borgatti EC, Baiesi Pillastrini F, Gabrielli L et al (2022) Universal Newborn Screening for Congenital Cytomegalovirus Infection - From Infant to Maternal Infection: A Prospective Multicenter Study. Front Pediatr 10:909646. 10.3389/fped.2022.909646 10.3389/fped.2022.909646 PMC9298552 35874574

[7] Yamamoto AY, Mussi-Pinhata MM, Marin LJ, Brito RM, Oliveira PF, Coelho TB (2006). Is saliva as reliable as urine for detection of cytomegalovirus DNA for neonatal screening of congenital CMV infection?. J Clin Virol.

[8] Gantt S, Goldfarb DM, Park A, Rawlinson W, Boppana SB, Lazzarotto T et al (2020) Performance of the Alethia CMV Assay for Detection of Cytomegalovirus by Use of Neonatal Saliva Swabs. J Clin Microbiol 58(4). 10.1128/JCM.01951-19 10.1128/JCM.01951-19 PMC7098765 31969426

[9] Shlonsky Y, Smair NS, Mubariki R, Bamberger E, Hemo M, Cohen S et al (2021) Pooled saliva CMV DNA detection: A viable laboratory technique for universal CMV screening of healthy newborns. J Clin Virol 138:104798. 10.1016/j.jcv.2021.104798 10.1016/j.jcv.2021.104798 33770655

[10] Alethia-LAMP-CMV assay, Meridian Bionscience. https://www.meridianbioscience.com/diagnostics/disease-areas/pediatric-neonatal/cmv/alethia-cmv/. Accessed 20 Jun 2023

[11] TIB Molbiol LightMix. https://www.tib-molbiol.de/products-/-solutions/kits . Accessed 20 Jun 2023

[12] Luck SE, Wieringa JW, Blázquez-Gamero D, Henneke P, Schuster K, Butler K (2017). Congenital Cytomegalovirus: A European Expert Consensus Statement on Diagnosis and Management. Pediatr Infect Dis J.

[13] Izquierdo G, Sandoval A, Abarzua F, Yamamoto M, Rodriguez JG, Silva M (2021). Recommendations for the diagnosis and management of cytomegalovirus infection in pregnant woman and newborn infant. Rev Chilena Infectol.

[14] Lorenzoni F, Lunardi S, Liumbruno A, Ferri G, Madrigali V, Fiorentini E (2014). Neonatal screening for congenital cytomegalovirus infection in preterm and small for gestational age infants. J Matern Fetal Neonatal Med.

[15] Fowler KB, McCollister FP, Sabo DL, Shoup AG, Owen KE, Woodruff JL et al (2017) A Targeted Approach for Congenital Cytomegalovirus Screening Within Newborn Hearing Screening. Pediatrics 139(2). 10.1542/peds.2016-2128 10.1542/peds.2016-2128 PMC5260148 28049114

[16] Purswani MU, Russell JS, Dietrich M, Malee K, Spector SA, Williams PL et al (2020) Birth Prevalence of Congenital Cytomegalovirus Infection in HIV-Exposed Uninfected Children in the Era of Combination Antiretroviral Therapy. J Pediatr 216:82–7 e2. 10.1016/j.jpeds.2019.09.025 10.1016/j.jpeds.2019.09.025 PMC6930703 31668479

[17] Hailemariam M, Mekonnen Z, Nys E, Padalko E (2021). (2021) Sample stability for congenital cytomegalovirus assay using Alethia after prolonged storage at different temperatures. GSC Advanced Research and Reviews.

[18] MagDEA Dx SV. https://www.pss.co.jp/english/product/reagent/magdea1.html. Accessed 22 Jun 2023

[19] TIB Molbiol LightMix. https://www.tib-molbiol.de/products-/-solutions/kits. Accessed 22 Jun 2023

[20] GeneProof Cytomegalovirus^®^ PCR Kit. https://www.geneproof.com/geneproof-cytomegalovirus-cmv-pcr-kit/p1081. Accessed 22 Jun 2023

[21] Boppana SB, Ross SA, Shimamura M, Palmer AL, Ahmed A, Michaels MG (2011). Saliva polymerase-chain-reaction assay for cytomegalovirus screening in newborns. N Engl J Med.

[22] Fernandes C, Marques A, de Jesus CM, Braz MC, Ferreira AR, Neto AS (2021). Saliva pools for screening of human cytomegalovirus using real-time PCR. Eur J Pediatr.

[23] Silva J, Fernandes C, Marques A, Maria AT, Correia C, Tuna ML et al (2020) Evaluation of saliva pools method for detection of congenital human cytomegalovirus infection. J Virol Methods 275:113759. 10.1016/j.jviromet.2019.113759 10.1016/j.jviromet.2019.113759 31678048

